# Cinnamon Leaf and Clove Essential Oils Are Potent Inhibitors of *Candida albicans* Virulence Traits

**DOI:** 10.3390/microorganisms10101989

**Published:** 2022-10-08

**Authors:** Zinnat Shahina, Ali Molaeitabari, Taranum Sultana, Tanya Elizabeth Susan Dahms

**Affiliations:** Department of Chemistry and Biochemistry, University of Regina, Regina, SK S4S 1P4, Canada

**Keywords:** antifungals, anti-virulence, *Candida albicans*, *Cinnamomum zeylanicum*, *Eugenia caryophyllus*, plant-based essential oils, synergism

## Abstract

Plant-based essential oils are promising anti-virulence agents against the multidrug-resistant opportunistic pathogen *Candida albicans*. Gas chromatography–mass spectrometry of *Cinnamomum zeylanicum* (cinnamon) leaf and *Eugenia caryophyllus* (clove) flower bud essential oils revealed eugenol (73 and 75%, respectively) as their major component, with β-caryophyllene, eugenyl acetate, and α-humulene as common minor components. Cinnamon leaf and clove essential oils had minimum inhibitory concentrations of 600 and 500 µg/mL, respectively against the *C. albicans* RSY150 reference strain and 1000 and 750 µg/mL, respectively for the clinical reference strain ATCC 10231. The combined oils are additive (FICI = 0.72 ± 0.16) and synergistic (0.5 ± 0.0) against RSY150 and the clinical reference strain, respectively. Mycelial growth was inhibited by sublethal concentrations of either essential oil, which abolished colony growth. At half of the lowest combined lethal concentration for the two oils, the yeast-to-hyphal transition and mycelial growth was potently inhibited. Mutant strains *als1*Δ/Δ, *als3*Δ/Δ, *hwp1*Δ/*HWP1*+, and *efg1*Δ/Δ were sensitive to either or both oils, especially *efg1*Δ/Δ. In conclusion, oils of cinnamon leaf and clove and their combination significantly impact *C. albicans* virulence by inhibiting hyphal and mycelial growth.

## 1. Introduction

*C. albicans*, a commensal organism that occupies the skin, mucosa, and gastrointestinal tract of healthy individuals, can cause mucosal, superficial, or life-threatening systemic infections in immunocompromised individuals, with a 40% mortality rate [1,2]. The increase in fungal resistance, the growing number of older patients, the continual emergence of clinically aggressive strains [3], and tolerance to existing antifungal drugs have elevated the spread of infection, warranting the search for effective therapies. Virulence factors are key to *C. albicans* pathogenicity, particularly dimorphism, the rapid switching between yeast and hyphal forms in response to environmental conditions. Other known virulence attributes include adhesion, proteolytic enzyme secretion, rapid filamentation, hyphal growth, biofilm formation, and invasion, all of which support fungal colonization and infection [4]. 

Adhesion and the subsequent transition to the hyphal form, a crucial step to initiate infection [5], are accompanied by the upregulation of adhesion-specific genes belonging to the agglutinin-like sequence (*ALS*) family (1–9), the hyphal wall protein 1 (*hwp1*), and their upstream transcription factor, enhanced filamentous growth protein 1 (*efg1*). Furthermore, the secretion of extracellular hydrolytic enzymes aids in subsequent host invasion [6]; for example, phospholipase and proteases degrade the host epithelial layer, allowing candida to disseminate. These crucial virulence mechanisms contribute to the establishment of persistent infections that are resistant to classical antifungals. 

Since fungi, like human cells, are eukaryotes, classical antifungals have targeted processes unique to fungi, including cell wall biosynthesis and ergosterol, but are subject to decreased efficacy, toxicity, and increased tolerance [7,8]. An emerging trend in developing novel and effective antifungals is to target fungal virulence mechanisms, both for prophylaxis and therapy of topical and systemic infections [9,10]. An array of plant-derived organic molecules are known to interfere with fungal virulence mechanisms [11,12], for example, certain secondary plant metabolites known as essential oils (EOs) extracted from the bud, flower, leaves, and bark of plants. Both clove (*Eugenia caryophyllus* or *Syzygium aromaticum* L.) and cinnamon (*Cinnamon zeylanicum*) essential oils are known for their antifungal and anti-virulence properties [11,13]. Their major constituents include polyphenols, alcohol, terpenes, aldehydes, terpenoids, and sesquiterpenes [14,15], with chemical compositions varying with growth environment (e.g. season, soil cultivation, etc.), plant parts, and extraction method, the latter including distillation, compression, and expression [14]. For example, the bark and leaves of *C. zeylanicum* have vastly different compositions, with cinnamaldehyde (65–85%) and eugenol (40–95%) as dominant components, respectively, and other major or minor components that contribute to their activity [16]. Eugenol is also the major component of clove oil, which shares minor components in varying concentrations with cinnamon leaf oil [17]. Several studies demonstrate the combined action of thymol, carvacrol, eugenol, menthol, and linalool [18,19] with conventional antifungal agents [20,21,22,23], but there are no reports on the combined effect of these essential oil components (EOCs) on *Candida* spp. The synergistic effects of EOCs have largely been studied in the context of bacteria, as reviewed by [19] who suggest that their combined antimicrobial mechanisms may relate to the sequential inhibition of a common biochemical pathway or the inhibition of protective enzymes, resulting in cell membrane damage that ultimately enhances uptake. 

Cinnamon bark oil induces cell wall remodeling by modulating its biosynthetic enzymes and alters membrane permeability by interfering with ergosterol pathways [24,25]. Cinnamon bark oil and its major component, cinnamaldehyde, exhibit strong antifungal activity and alter cell wall and membrane integrity, ultimately causing cell cycle arrest [24]. Although fungicidal activity has been reported for cinnamon leaf oil extract from *Cinnamomum* spp., the associated mechanisms remain unclear [26]. Clove oil and its major component, eugenol, are known for their fungicidal and anti-virulence properties, including cell membrane compromise, modified nutrient transport, cell cycle arrest, and inhibition of germ tube and biofilm formation [11,17,27,28,29]. Since both EOs share eugenol as their major component, we hypothesized that differences in the minor components of cinnamon leaf and clove bud oil would lead to altered antifungal strengths and differential impacts on virulence factors.

This study examines the impact of cinnamon leaf (CNL) oil from *Cinnamomum zeylanicum* and clove flower bud (CLV) oil from *Eugenia caryophyllus* alone and in combination, and how they impact *C. albicans* virulence traits including hyphal morphogenesis, mycelial growth, secretion of hydrolytic enzymes, and the sensitivity of strains having key virulence gene knockouts. 

## 2. Materials and Methods

### 2.1. Essential Oils and Other Chemicals

Steam distillates of cinnamon leaf, registry number 8015-91-6, and clove essential oils were from *Cinnamon zeylanicum* leaves and *Eugenia caryophyllus* flower buds (Aroma Force, A. Vogel, Dollard-des-Ormeaux, QC, Canada), respectively. Amp B (amphotericin B), fetal bovine serum, CaCl_2_, glucose, K_2_HPO_4_, NaCl, mannitol, nutrient broth, phosphate-buffered saline powder (0.01 M phosphate pH 7.4; 0.138 M NaCl, 0.0027 M KCl in 1 L ultrapure water; PBS), Sabouraud dextrose agar (SDA), bovine serum albumin (BSA) were purchased from Sigma Aldrich Chemical Co. (St. Louis, MO, USA). Bacto^TM^ agar, yeast extract, and peptone were purchased from Difco (BD Biosciences, Franklin Lakes, NJ, USA). 

### 2.2. Strains and Cultural Conditions

The strains used in this study are listed in Table S1. The laboratory strain *C. albicans* RSY150 (*TUB2-GFP-SAT1/TUB2*_ *HTB1-RFP-ARG4*_/*HTB1*_ *arg4*) was a gift from Dr. Richard J. Bennett (Department of Molecular Microbiology and Immunology, Brown University, RI, USA), while *C. albicans* clinical strains, including the ATCC 10231 clinical reference, blood, genital, and fluconazole resistant isolates were provided through collaboration with the Regina Qu’Appelle Health Region (Department of Microbiology, Regina General Hospital, Regina, SK, Canada). Knockout strains *hwp1*Δ/*HWP1*^+^, and *efg1*Δ/Δ, *als1*Δ/Δ and *als3*Δ/Δ were a kind gift from Dr. Malcolm Whiteway (Department of Biology, Concordia University, PQ, Canada) and Dr. Lois L. Hoyer (Department of Veterinary Pathobiology, University of Illinois at Urbana-Champaign, Urbana, IL, USA), respectively.

Strains were stored as 50% glycerol stocks at −50 °C and were freshly revived on yeast-extract peptone dextrose (YPD) agar containing 1% Bacto-yeast extract, 2% Bacto-peptone, 2% glucose, and 2% Bacto-agar prior to each experiment. Strains were grown with continuous shaking at 30 °C in YPD broth. For the yeast-to-hyphal transition and leakage assays, cells were grown to mid-log phase prior to essential oil exposure. To induce hyphae, cells were grown in YPD broth with 10% fetal bovine serum and 2% glucose. For all assays, unless otherwise stated, blanks consisted of the essential oils in PBS, growth controls were untreated cells in YPD, and the positive control was Amp B at MIC. 

### 2.3. Gas Chromatography (GC)–Mass Spectrometry (MS) Analysis

Essential oil composition was analyzed by gas chromatography flame ionization detection (GC-FID) and gas chromatography–mass spectrometry (GC–MS) using an Agilent 7890A GC. Analysis conditions, instrumentation, and compound identification are described in [24].

### 2.4. Minimum Inhibitory Concentration (MIC) and Fractional Inhibitory Concentration Index (FICI)

The MIC of CNL and CLV oils against *C. albicans* was determined according to the Clinical and Laboratory Standards Institute guidelines [30] and reported methods [31] using working solutions of 8000 µg/mL CNL and 7600 µg/mL CLV. Culture conditions, measurements, and data analysis are described in our previous study [24]. 

The fractional inhibitory concentration index, used to assess the interaction between CNL and CLV oils, was determined using a checkerboard assay as previously reported [32], but with slight modification. Serial dilutions (2.22-fold) of CNL and CLV in YPD were prepared within the MIC range with 90 μL of CNL and CLV solution added to rows and columns, respectively, of 96-well microtiter plates in decreasing concentrations. A 20 μL aliquot of *C. albicans* culture (2.2 × 10^5^ cells/mL) was added to each test well of the microtiter plate, with growth controls containing YPD only and blanks having YPD and EO(s) without *C. albicans*. The microtiter plates were incubated with shaking at 30 °C for 24 h and the OD_600_ was recorded using a BioTek Synergy HTX microplate reader (Northern Vermont, USA). The MIC was determined as the first well with a growth reduction of 100% in the presence of the EO in comparison to the growth control. The MIC from the checkerboard assay was used to calculate the FIC and FICI according to the following formulae:

(1) $$\mathrm{FIC}\;\left(\mathrm{CNL}\right)=\frac{\mathrm{MIC}\;\mathrm{of}\;\mathrm{CNL}\;\mathrm{in}\;\mathrm{combination}}{\mathrm{MIC}\;\mathrm{of}\;\mathrm{CNL}\;\mathrm{alone}}\;$$

(2) $$\mathrm{FIC}\;\left(\mathrm{CLV}\right)=\frac{\mathrm{MIC}\;\mathrm{of}\;\mathrm{CLV}\;\mathrm{in}\;\mathrm{combination}}{\mathrm{MIC}\;\mathrm{of}\;\mathrm{CLV}\;\mathrm{alone}}$$

(3) FICI=FIC of CNL+FIC of CLV

**Figure 1:**
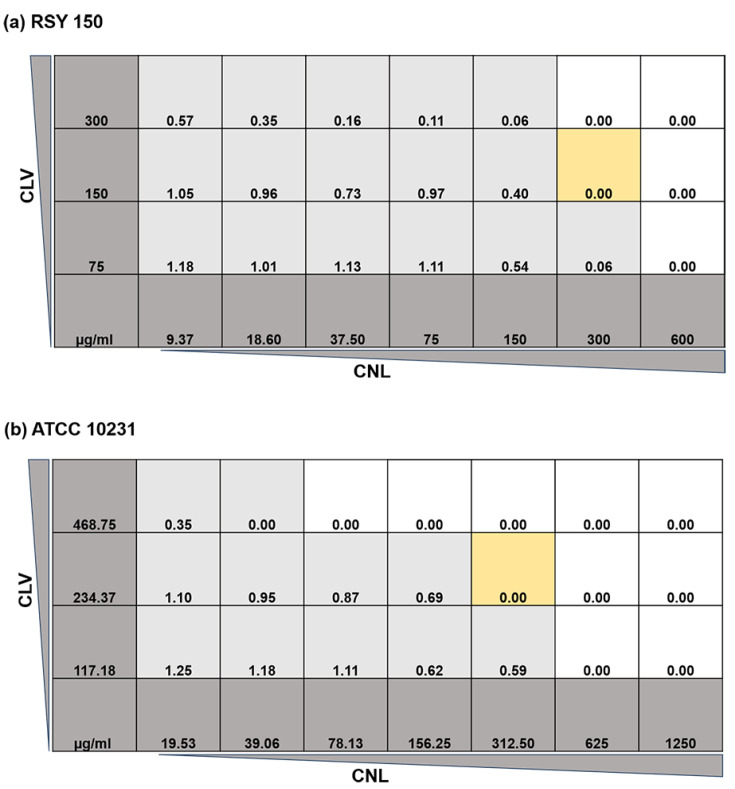
Checkerboard microtiter plate assay of *C. albicans* (**a**) RSY150 and (**b**) ATCC10231. Dark grey is oil concentration, light grey is visible growth, white is no growth, and light brown highlights the lowest combined lethal concentration (LCLC) having zero growth (OD_600_).

Based on the index, the combined activity is considered synergistic (FICI ≤ 0.5), additive/partially synergistic (0.5 < FICI ≤ 1), indifferent (1 < FICI ≤ 4.0), or antagonistic (FICI > 4.0) [32]. In the figures and tables, we refer to the fractional (1/8–1/2) lowest combined lethal concentrations (LCLC) of the two components (see Figure 1). 

### 2.5. Membrane Leakage Assays

A cellular content leakage assay was used to assess the impact of CNL and CLV oils on *C. albicans* RSY150 cell membrane integrity according to a previously reported method [33], with slight modification. Briefly, *C. albicans* RSY150 at mid-log phase were washed three times and resuspended to ~10^7^ CFU/mL in PBS, then exposed to EOs alone or in combination in 96-well micro plates. The plates were then incubated at 30 °C for 6 h with continuous shaking (200 rpm). EOs in PBS served as blanks, untreated cells in PBS served as negative controls, and Amp B at MIC served as a positive control. Following incubation, the supernatant was diluted 1:10 with PBS, filtered (0.22 μm), and the leakage of cellular materials was recorded as the absorbance at 260 nm (A_260,_ Varian Cary 100 BIO, UV–VIS spectrophotometer; Midland, ON, Canada). 

### 2.6. Propidium Iodide Uptake Assay

Propidium iodide (PI) was used to identify *C. albicans* RSY 150 cell death according to the previously described method [34,35], with slight modification. Briefly, following a 4-h treatment of *C. albicans* in a 24-well plate with EOs or Amp B 1/2 and full MIC, cell density was normalized to 10^5^ cells/mL, 200 µL of the suspension transferred to the wells of a flat-bottom 96-well microplate to which 2 µL of PI (1 µg/mL, Sigma-Aldrich, St. Louis, MO, USA) was added, followed by a 30 min incubation at 30 °C in the dark. *C. albicans* were pipetted onto clean microscope slides, covered with a clean coverslip sealed with nail polish and imaged by epifluorescence (λ_ex_ = 493 nm; λ_em_ = 636 nm) with an Axio Observer Z1 inverted microscope (Oberkochen, Germany) and the fluorescence intensity measured with ZEN software (version 2.3) from a total of 50 individual cells each for two biological replicates imaged in ten microscopic fields of view.

### 2.7. Yeast-to-Hyphal Transition Assay

Serum-induced germ tube induction for *C. albicans* RSY 150 and ATCC 10231 was assessed in the presence of CLV and CNL EOs alone and together, according to a previously reported method [36]. Briefly, a yeast suspension (1 × 10^7^ CFU/mL) of mid-log phase cells in pre-warmed YPD with 10% fetal bovine serum (FBS) was deposited into a 12-well plate with the appropriate amounts of EOs to achieve MIC, 1/2 MIC, and 1/2 LCLC and incubated for 4 h at 37 °C. Following treatment, the cells were washed, centrifuged, and stained with calcofluor white (0.01 μg/mL, Fluka Analytical, New Delhi, India) to highlight the hyphal and pseudo-hyphal cell wall and septa. Approximately 5 μL of treated or untreated *C. albicans* suspended in PBS were mounted on sealed microscope slides and imaged by epifluorescence. The percentage of germ tubes, hyphae, or pseudohyphal forms were quantified from images. Germ tubes were counted when the size of the cell projection was equivalent to that of the blastospore. Results from three biological replicates were expressed as GTF/300 cells (germ tube forming cells per 300 cells counted). 

### 2.8. Mycelial Growth Assay

Spider media agar plates (1% nutrient broth, 1% mannitol, and 0.2% K2HPO4) with or without CNL or CLV at 1/2 MIC, 1/4 MIC, or the two 1/2 LCLC were used to assess impacts on mycelial growth. Aliquots (2 μL) of a *C. albicans* RSY150 suspension (~1 × 10^7^ cfu/mL) at mid-logarithmic phase were spotted onto spider agar media in a 35 mm polystyrene cell culture dish (Sarstedt, Nümbrecht, Germany) according to published protocols [36,37]. The plates were incubated for 6 days at 37 °C and the presence or absence of colony wrinkles were observed and imaged with a digital camera. 

### 2.9. Phospholipase and Proteinase Activity Assay

Phospholipase (P_z_) and proteinase (P_rz)_ activity was assessed according to published protocols [36,38]. For the P_z_ assay, SDA (65 g SDA, 58.4 g NaCl and 5.5 g CaCl_2_) with 2% egg yolk (supernatant added to cooled (45–50 °C) medium following centrifugation (5000× *g*, 30 min)) was mixed and dispensed as a test medium, whereas proteinase activity (P_rz_) was evaluated using BSA as a substrate in yeast agar (11.7 g glucose, 0.1 g yeast extract, and 20 g agar in 1 L distilled water with 2% BSA, pH 5.0). P_z_ and P_rz_ activity were assessed for *C. albicans* pre-treated with EOs 1/2 MIC or the two 1/2 LCLC. Cultures of both *C. albicans* strains at mid-logarithmic phase (10^7^ cfu/mL) were treated with or without EOs for 4 h, washed with PBS, and 5 μL was spotted onto media plates. Pz and Prz activity was calculated according to colony diameter and the zone of clearance after 4 d incubation at 37 °C, according to the following formula [38].

$$\mathrm{Pz}/\mathrm{Prz}=\frac{\mathrm{Colony}\;\mathrm{diameter}}{\mathrm{Colony}\;\mathrm{diameter}+\mathrm{Zone}\;\mathrm{of}\;\mathrm{clearance}}$$

Enzyme activity was scored accordingly, where 1 indicates no enzyme activity, a value of <0.90–0.99 is low (+), 0.80–0.89 is medium (++); 0.70–0.79 is strong (+++) and <0.70 is very strong (++++).

### 2.10. Statistical Analyses

All experiments were conducted on three biological replicates, each in triplicate unless otherwise indicated, and GraphPad Prism (Version 6.0; La Jolla, CA, USA) was used for statistical analyses. All results are reported as the mean ± standard deviation (SD) of the biological replicates, and differences were assessed using a two-tailed unpaired *t* test with Welch’s correction at a 95% confidence interval, for which *p* < 0.05 was considered statistically significant.

## 3. Results

### 3.1. CNL and CLV Essential Oils Share Similar Major Components

GC-FID and GC–MS analysis of the CNL and CLV EOs revealed several identical components (Table 1a,b; Figure S1). Twenty-four components were detected in CNL, including the major component eugenol (73%) and other constituents such as β caryophyllene (4.8%), linalool (2.5%), benzyl benzoate (2.4%), a-copaene (2.4%), E-cinnamyl acetate (2.3%), and eugenyl acetate (2%), whereas eight components were identified in CLV oil, again with eugenol (75%) as the major component, along with β-caryophyllene (11.7%), eugenyl acetate (9.9%), and humulene (2%). Interestingly, all the compounds identified in CLV are also found CNL, but at different concentrations. 

### 3.2. CNL and CLV Are Additive and Synergistic against C. albicans

CNL and CLV oils have MICs in the range of 500–600 µg/mL and 470–600 µg/mL for *C. albicans* RSY150, and 1000 and 750–940 µg/mL for *C. albicans* ATCC 10231, respectively. Interestingly, the fluconazole-resistant strain was sensitive to CLV and CNL oils within the MIC range of the RSY150 reference strain (Table 2). Oils at 1/4 and 1/2 MIC were used for all subsequent experiments. There was some variation between the batches of oils but with similar MIC ranges and synergistic interactions (FICI) against *C. albicans* RSY150.

FICI values of CNL with CLV were 0.72 ± 0.16 and 0.5 ± 0.0 against RSY150 and ATCC 10231, respectively. The combination of CNL and CLV was additive against the lab RSY150 reference strain but consistently synergistic against the clinical reference strain ATCC 10231, with a 2.2-fold reduction of both MIC values compared to the substances alone. The checkerboard titer assay (Figure 1) also revealed the MIC of CNL alone to be 600 and 1250 μg/mL for the RSY and ATCC strains, respectively. The CNL MIC was lower, at 300 (RSY) and 312.5 (ATCC) μg/mL in the presence of CLV at 150 and 234.37 μg/mL, which alone had an MIC of 600 and 937.50 μg/mL for RSY and ATCC, respectively.

### 3.3. EOs Compromise Cell Membrane Integrity

**Figure 2:**
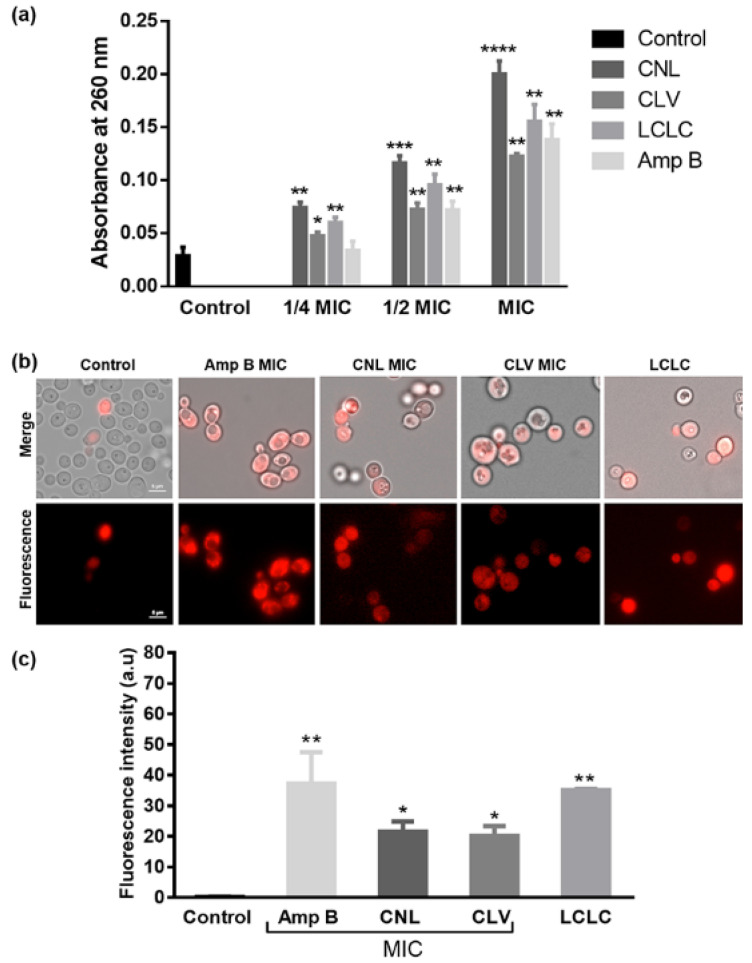
Membrane integrity assays of *C. albicans* RSY150 exposed to EOs. (**a**) CNL, CLV alone and together caused leaky membranes and subsequent cell death. (**b**) Representative merged bright-field/fluorescence (**top**) and fluorescence (**bottom**) images (λ_ex_ = 493 nm; λ_em_ = 636 nm) and (**c**) bar graphs of treated *Candida* show significant PI uptake as compared to controls. Scale bars are 5 μm for controls and applicable to all images. Untreated cells served as a negative control, and those treated with Amp B were used for comparison. Asterisks indicate statistically significant differences (****, *p* < 0.0001; ***, *p* = 0.0002; **, *p* = 0.001–0.0012, and *, *p* = 0.0419) compared to the untreated controls.

A nucleic acid leakage experiment was conducted to access the impact of exposure to the EOs and positive control Amp B on *C. albicans* RSY150 membrane permeability (Figure 2). Cellular content leakage increased in a dose-dependent manner from 1/4 MIC to MIC under all conditions (Figure 2).

*C. albicans* RSY150 controls (Figure 2b) had little PI fluorescence, but there was a significant (*p* < 0.01) signal with exposure to the positive control, Amp B at MIC, and the combined EOs at LCLC. On the other hand, exposure to CNL and CLV at MIC showed statistically less (*p* < 0.05) PI uptake (Figure 2c). This data shows that the permeability barrier is broken at high EO exposure, leading to cell death.

### 3.4. EOs Inhibit Germ Tube Formation in C. albicans RSY150

**Figure 3:**
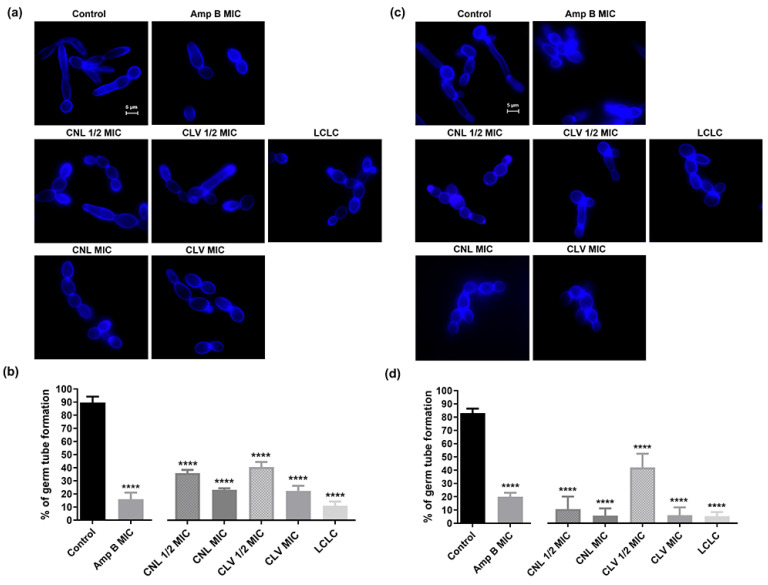
Effects of CNL, CLV, and Amp B on *C. albicans* germ tube formation. (**a**,**c**) Representative epifluorescence microscopy (λ_ex_ = 365 nm; λ_em_ = 435 nm) images following exposure of (**a**) RSY150 and (**c**) ATCC 10231 to EOs and Amp B for 4 h. (**b**,**d**) Bar graphs show the quantitative analysis of (**b**) RSY150 and (**d**) ATCC 10231 germ tube formation for 300 cells from three biological replicates. EOs at all concentrations tested significantly (****, *p* < 0.0001) inhibited the yeast-to-hyphal transition.

CNL and CLV at MIC, 1/2 MIC, and the two at their LCLC were potent inhibitors of the *C. albicans* RSY150 and ATCC 10231 yeast-to-hyphal transition (Figure 3a,c). The germ tube-positive *C. albicans* RSY150 cells had proportionately fewer germ tubes at MIC and 1/2 MIC, with a 4.8- and 2.4-fold decrease, respectively, in the presence of CNL and CLV. Likewise, ATCC 10231 had a 13.8-fold reduction of germ tubes in the presence of either CNL or CLV at 1/2 MIC, and 7.5- and 2-fold reductions with CLV and CNL treatment at MIC (Figure 3b,d). The most potent germ tube inhibitor (9.5-fold) was the combined EOs at LCLC, followed by CLV (4.5-fold) and CNL (4.3-fold) at their MICs. The positive control Amp B at its MIC also showed a significant (*p* < 0.0001) 5.9-fold reduction in germ tube formation.

### 3.5. CNL with CLV Inhibits C. albicans Mycelial Growth

Continuous exposure to CNL or CLV at 1/2 MIC, 1/4 MIC, and the two at 1/2 LCLC suppressed mycelial growth. *C. albicans* RSY150 grown on spider media in the presence of CNL or CLV had small, abnormally shaped colonies and a smooth surface, indicating total arrest of this morphological transformation, in direct contrast to untreated colonies that were highly wrinkled and uneven (Figure S2). The two at 1/2 LCLC had compromised viability with a 100% reduction of mycelial growth. 

### 3.6. EO Exposure Impacts Exoenzyme Secretion

Untreated *C. albicans* RSY150 was strongly positive (P_z_ < 0.70) for phospholipase and proteinase activity (Table 3), whereas 100% of those exposed to CLV and CNL at 1/2 MIC or the two at 1/2 LCLC had no detectable protease activity (P_z_ = 1). Phospholipase activity was inhibited by exposure to CLV at 1/2 MIC and the two at 1/2 LCLC, but not CNL at 1/2 MIC. The clinical background strain (ATCC 10231) was highly virulent (P_z_ < 0.7) for both phospholipase and protease activity. Upon exposure to CNL at 1/2 MIC or the two EOs at 1/2 LCLC, phospholipase activity was moderate (Pz = 0.70–0.79) but was not impacted by CLV, while proteinase activity was significantly inhibited by both EOs at 1/2 MIC (Table 3), for which CLV had a less pronounced effect (Pr_z_ = 0.83).

### 3.7. C. albicans Virulence Mutants have Differential Sensitivity to CNL and CLV

Mutants with gene knockouts of key virulence genes had differential sensitivity to CNL and CLV oils (Table 4; Table S2). For instance, the adhesin (*als1* and *als3*) knockout mutants were sensitive to CNL, while only the *als1* knockout was sensitive to CLV. Interestingly, the knockout mutant of transcription factor *efg1*, a strong regulator of morphogenesis in *C. albicans*, was sensitive to both CNL (~3-fold) and CLV (~2-fold) compared to the SC5314 background strain. The MIC values of wild type (SC5314) and mutant strains, from which sensitivity fold change were determined, are shown in Table S2. 

## 4. Discussion

*Candida* infections, common among immunocompromised patients, cause high morbidity and mortality [1,7,40,41]. There is a wealth of information on the pathogenesis of *Candida* infections, including the interplay of key virulence factors that protect it against a myriad of host defenses and antifungals [4,5]. With *Candida* exhibiting resistance to many classical antifungals, alternative prevention and treatment options are required, with a paradigm shift towards targeting virulence factors [9,10]. While the fungicidal and anti-virulence properties of plant-based essential oils from CLV, CNL, rosemary, lemon grass, and jasmine have been documented, the associated mechanisms are not well defined [13,14,17,24,27,36,42,43,44]. Herein we examine the anti-virulence activity of CLV and CNL oils, identify their key virulence gene targets, and assess their antifungal efficacy in combination.

Sublethal doses of both CNL and CLV essential oils altered membrane integrity, increased its permeability, and caused leakage of cellular components (Figure 2), consistent with the lipophilic nature of their components and previous studies [45]. The antifungal potency of cinnamon bark and CLV oils has been attributed to its major components cinnamaldehyde and eugenol, the latter shown to inhibit ergosterol biosynthesis, leading to membrane disruption and cell death [24,43], which is consistent with CNL and CLV oils sharing the major component eugenol (Table 1), an effective disruptor of membrane integrity (Figure 2). Membrane compromise initiates a series of events that impact a wide range of processes vital for infection, including the secretion of hydrolytic enzymes, endocytosis, hyphal morphogenesis, and filamentation, all of which lead to attenuation of virulence [46]. 

The yeast-to-hyphal transition is critical for virulence, with mutants lacking the ability to morphologically switch to hyphal forms [47,48]. Since both CNL and CLV effectively inhibited hyphal formation (Figure 3), with clinical strains having higher sensitivity than reference strains, these oils are promising future candidates for antifungal design and prophylaxis. Sublethal concentrations of eugenol can completely inhibit hyphal morphogenesis in reference and clinically relevant *C. albicans* strains, along with dermatophyte species [27], while the minor component of CNL and CLV oil, eugenyl acetate, is a potent inhibitor of both germ tube and biofilm formation [49]. Thus, the two-fold reduction in germ tube formation (Figure 3) with CNL/CLV at half their lethal combination mostly likely reflects the contribution of minor components, for example, β-caryophyllene/eugenyl acetate (Table 1). 

Hyphal morphogenesis also leads to a wide range of cellular processes, including hydrolytic enzyme production and expression of surface proteins such as adhesins and invasins [47,50]. *C. albicans* secretes hydrolytic lipases, phospholipases and aspartic proteases (Saps) that damage host epithelial surfaces and initiate invasion [6]. Extracellularly secreted phospholipases act together with proteases, both secreted and membrane-bound, to cause host cell membrane disruption [6]. The proteases, highly expressed in the reference and clinical strains, were inhibited equally by CNL and CLV oils (Table 3). The highly virulent clinical strain, which secreted a large amount of phospholipases in comparison to the RSY150 reference strain, was moderately impacted by CNL and CLV. On the other hand, the phospholipases of reference strains had differential inhibitory responses to the oils, with moderate inhibition by CLV or the combined CNL/CLV, and no impact by CNL, consistent with CLV having moderate inhibitory activity in comparison to rosemary oil, and the greater impact of cinnamaldehyde in comparison to eugenol [42]. 

Hyphal morphogenesis is directly or indirectly intertwined with the expression of cell surface adhesin proteins, which include the hyphal wall protein (Hwp1) [51] and the Als family (1–9) of proteins that are involved in both initial adhesion and in maintaining adhesion following biofilm formation [52,53,54]. Among the Als members, Als1/Als3 are mainly involved in initial attachment events and have functional redundancy with Hwp1. Als1/Als3 have been shown to interact with Hwp1 to facilitate adhesion between cells and maintain biofilm cohesion [52]. Interestingly, both cellular morphology and the expression of those genes are tightly regulated by transcriptional activators and repressors such as Efg1, Tec1, Bcr1, Tup1, and Nrg1 [55], and some adhesion-deficient *C. albicans* strains (*efg1*Δ and *bcr1*Δ) show normal initial adhesion but defective adhesion maintenance [56]. Indeed, *in vitro* and *in vivo* studies of *C. albicans* exposed to curcumin, pyrocatechol [57], linalool [58], quinic acid [59], and eugenol tosylate [60] show limited regulation of many of these genes. Mutations in *hwp1* and *als3* reduce hyphal and biofilm formationand, *C. albicans* with cell wall assembly defects are usually less virulent or avirulent [61,62,63]. The mutant strains *efg1Δ*, *als1Δ, als3Δ,* and *hwp1Δ* were sensitive to CNL and CLV (Table 4), implying that these cell wall proteins confer *C. albicans* tolerance to the EOs. Since the cell membrane plays a critical role in virulence, by mediating the secretion of virulence factors, orchestrating endocytosis, cell wall synthesis, and invasive hyphal morphogenesis (Douglas 2016), it is possible that relatively minute changes to the cell membrane structural integrity detrimentally impact the secretion of virulence proteins. As Efg1 plays a major role in the transcriptional regulation of the *als* and *hwp* genes, and since there is a link between its regulation of Hwp1 [64] and biofilm formation [65], the greater sensitivity of the *efg1*Δ/Δ mutant to CNL compared to CLV is of future interest. 

**Figure 4:**
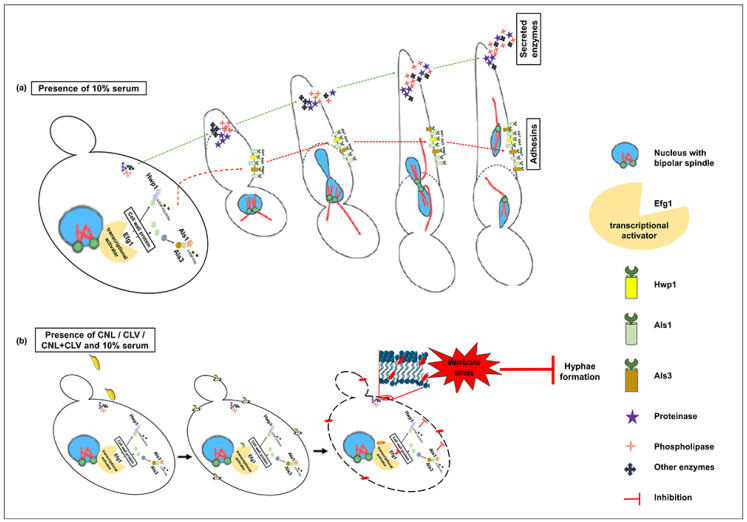
Model of the impact of CNL and CLV on *C. albicans* hyphal development. (**a**) Serum normally triggers the yeast-to-hyphal transition at 37 °C, which requires transcription factors such as Efg1 that play a major role in the expression of cell wall adhesion proteins (Hwp1, Als1, Als3, etc.). Subsequent hyphal elongation involves Hgc1, Eed1, and Ume6, of which Eed1 and Ume6 are negatively regulated by Tup1 and Nrg1 [66]. Hyphal initiation enables hydrolytic enzyme secretion, including proteinases (Sap), phospholipase B enzymes, and lipases, which play an important role in the pathogenicity of candidiasis. (**b**) At sublethal levels, we propose that the lipophilic EOs cause membrane stress that impacts cell wall proteins and secreted enzymes, thereby perturbing hyphal development.

Like past studies, we observe *C. albicans* lethality at higher CNL and CLV exposures, which can be attributed to significant membrane permeability and subsequent leakage of important cellular components (Figure 2),. However, at sublethal concentrations, membrane stress appears to impact membrane proteins and secreted enzymes (e.g., proteinases), which are important for hyphal development (Figure 4), leading to less myceliated colonies (Figure S2).

Overall, CNL had a greater impact on membrane integrity and germ tube formation than CLV, which can be explained by compositional differences (Table 1). Although the major component of both CNL and CLV oil is eugenol, they share several minor components such as β-caryophyllene, eugenyl acetate, and α-humulene, but in different proportions. Furthermore, CNL oil has many additional components such as linalool, benzyl benzoate, α-copaene, and E-cinnamyl acetate, which must account for the observed differences in their virulence attenuation. Although the anticandidal effects of the oils are attributable to the common major component, eugenol, the synergistic interaction between two or more components must influence anti-virulence activity, which is enhanced only when the oils are used in combination.

## Figures and Tables

**Table 1:** Chemical composition of essential oils (major component in bold).

(a) CNL				
Compound	% Yield	Exp RI ^a^	Lit RI ^a^	ID Method ^b^
α-pinene	1	935	932	MS
camphene	0.4	950	946	MS
benzaldehyde	0.3	961	952	RI, MS
β-pinene/sabinene *	0.3	979	974	MS
α-phellandrene	1.2	1007	1002	RI, MS
α-terpinene	0.1	1018	1014	RI, MS
p-cymene	0.9	1026	1020	RI, MS
limonene	0.7	1031	1025	MS
terpinolene	0.1	1090	1086	RI, MS
linalool	2.5	1102	1095	RI, MS
α-terpineol	0.2	1193	1186	RI, MS
E-cinnamaldehyde	1.3	1277	1267	MS
cinnamyl alcohol	0.2	1314	1259	MS
**eugenol**	**73**	**1376**	**1361**	**MS**
α-copaene	2.4	1383	1345	MS
β-caryophyllene	4.8	1428	1412	MS
E-cinnamyl acetate	2.3	1450	1443	RI, MS
α-humulene	0.8	1461	1452	MS
viridiflorene	0.2	1501	1471	MS
δ−χαδινενε	0.2	1529	1501	MS
eugenyl acetate	2	1534	1521	RI, MS
caryophyllene oxide	0.4	1591	1582	MS
benzyl benzoate	2.4	1772	1759	RI, MS
Total	97.7			
**(b) CLV**				
**Compound**	**% Yield**	**Exp RI ^a^**	**Lit RI ^a^**	**ID Method ^b^**
**eugenol**	**75**	**1378**	**1361**	**RI, MS**
methyl eugenol	0.2	1406	1403	MS
β-caryophyllene	11.7	1416	1412	RI, MS
α-humulene	2	1451	1446	RI, MS
δ−χαδινενε	0.2	1522	1518	RI, MS
eugenyl acetate	9.9	1529	1526	RI, MS
caryophyllene oxide	0.4	1580	1573	RI, MS
Total	99.4			

^a^ RI = Retention index on a HP-5 column with reference to n-alkanes [39]; Estimated error: ±0.1 % (area) and ± 1 for RI between trials; Lit = Literature, Exp = Experimental; ^b^ MS = mass spectrometric analysis; The relative area under the peak was estimated and experimental data compared with MS, NIST library spectra and the literature [40,41]; * cannot distinguish with RI or MS.

**Table 2:** Relative sensitivity of *C. albicans* ATCC 10231, RSY150 and clinical isolates.

	MIC Range (µg/mL)	
*C. albicans* Strains	CNL	CLV
RSY150	500–600	469–600
ATCC 10231	1000–1250	750–938
Genital Specimen	625–1000	750–938
Blood Culture	625–1000	750–938
Fluconazole Resistant Genital Specimen	500–625	469–750

**Table 3:** Inhibition of *C. albicans* RSY150 and ATCC 10231 enzymatic activity by EOs.

Enzyme Activity	Control	CNL 1/2 MIC	CLV 1/2 MIC	CNL+CLV(1/2) ^‡^
*C. albicans* RSY150				
Phospholipase (P_z_)	0.86 ± 0.02	0.89 ± 0.04	1.00 ± 0.01 *	1.00 ± 0.02 *
Proteinase (Pr_z_)	0.32 ± 0.05	1.00 ± 0.01 *	1.00 ± 0.03 *	1.00 ± 0.05 *
***C. albicans* ATCC 10231**				
Phospholipase (P_z_)	0.65 ± 0.05	0.76 ± 0.00 *	0.70 ± 0.03	0.73 ± 0.01 *
Proteinase (Pr_z_)	0.56 ± 0.05	1.00 ± 0.01 *	0.83 ± 0.03 *	1.00 ± 0.02 *

^‡^ CNL (150, 156.24 μg/mL) combined with CLV (75, 117.18 μg/mL) is half the LCLC for RSY150 and ATCC10231, respectively. * *p* < 0.05.

**Table 4:** Sensitivity of *C. albicans* SC5314, *als1*Δ/Δ, *als3*Δ/Δ, *hwp1*Δ/*HWP1*^+^ and HLC67(*efg1*Δ/Δ) to EOs and Amp B.

*C. albicans* Strains	CNL	CLV	Amp B
*als1*Δ/Δ (1467)	NC	+	NC
*als3*Δ/Δ (1843)	+	NC	NC
*hwp1*Δ/*HWP1*+	++	++	NC
HLC67(*efg1*Δ/Δ)	+++	++	+

NC, no change; + (~1 fold), ++ (~2 fold), +++ (~3 fold) 2.

## Data Availability

Data are accessible through the University of Regina data management archive.

## Supplementary Materials

The following supporting information can be downloaded at: https://www.mdpi.com/article/10.3390/microorganisms10101989/s1, Figure S1. GC–MS chromatogram of CNL and CLV EOs; Figure S2. Inhibition of hyphal morphogenesis of *C. albicans* RSY150 in the presence of CNL, CLV (1/2 MIC and 1/4 MIC) and the two at 1/2 LCLC on spider media agar plates. Images were recorded using a digital camera; Table S1. List of strains and clinical isolates used in this study; Table S2: MIC values of EOs against *C. albicans* SC5314, *als1*Δ/Δ, *als3*Δ/Δ, *hwp1*Δ/*HWP1*^+^ and HLC67(*efg1*Δ/Δ). References [47,67,68,69,70] have been cited in the Supplementary Materials.
