# Supplementary material for: Cinnamon Leaf and Clove Essential Oils Are Potent Inhibitors of Candida albicans Virulence Traits
Source: Microorganisms. 2022 Oct 8;10(10):1989. doi: 10.3390/microorganisms10101989 (PMC9607542; doi:10.3390/microorganisms10101989)
Supplement: Supplementary file 1 [file microorganisms-10-01989-s001.zip › microorganisms-1772725-supplementary.pdf]

Cinnamon leaf and clove essential oils are potent inhibitors of *Candida albicans* virulence traits

Zinnat Shahina, Ali Molaeitabari, Taranum Sultana, Tanya E. S. Dahms

Supplementary data

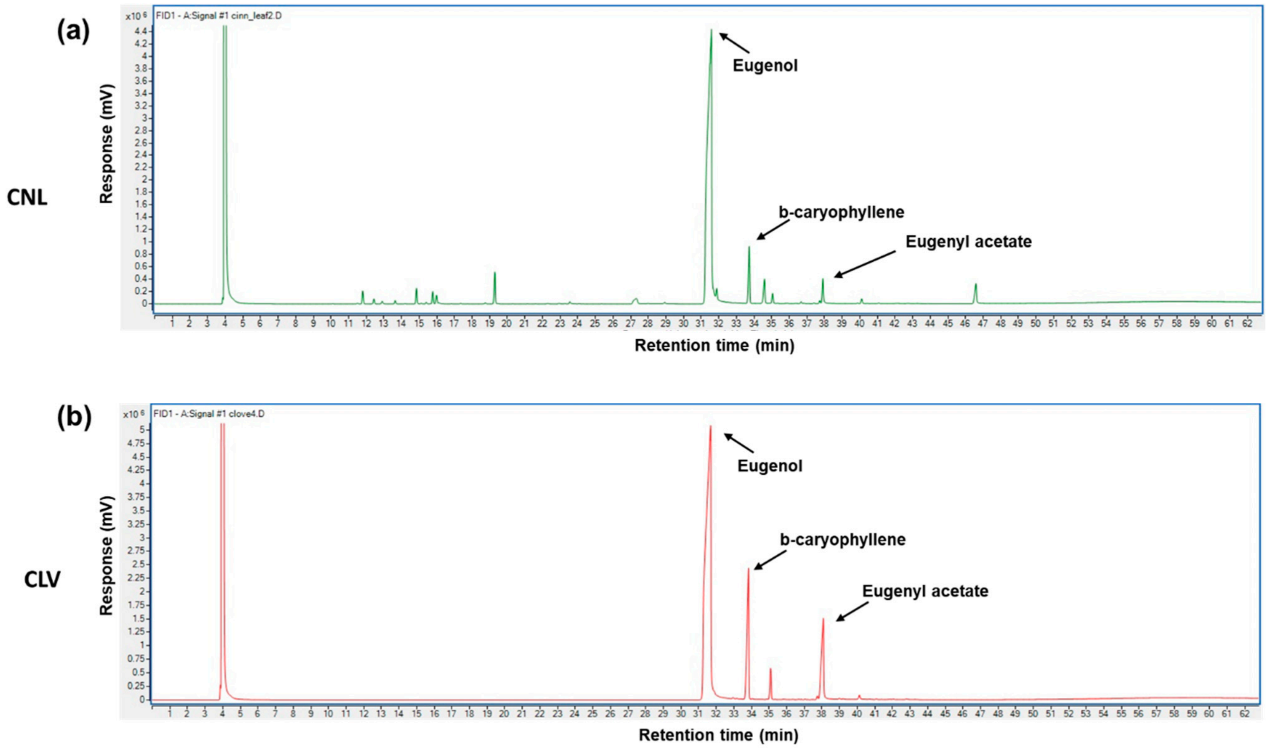

Figure S1.GC-MS chromatogram of CNL and CLV EOs.

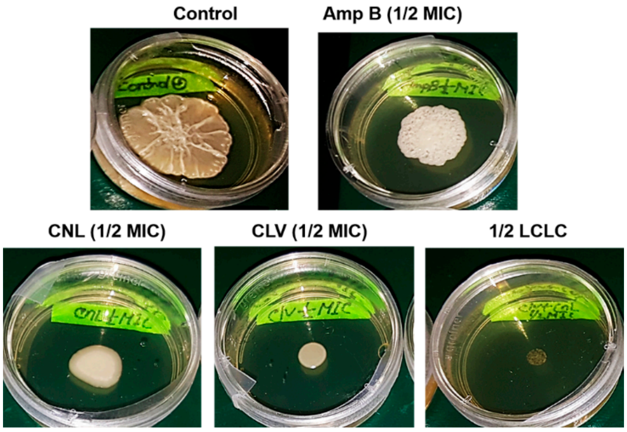

Figure S2. Inhibition of hyphal morphogenesis of *C. albicans* RSY150 in the presence of CNL, CLV (1/2 MIC and 1/4 MIC) and the two at 1/2 LCLC on spider media agar plates. Images were recorded using a digital camera.

Table S1. List of strains and clinical isolates used in this study.

**Strains**	**Genotype/ Isolate description**	**References**
RSY150[a]	*TUB2-GFP-SAT1/TUB2⁺ HTB1-RFP-ARG4⁺/HTB1⁺ arg4⁻/⁻*	[1]
ATCC 10231	Clinical background strain	RQHR[b], Regina SK, Canada
SC5314	Parent strain for knock out mutant below	[2]
C. albicans (1 – 3)	Clinical isolates; 2 genital and 1 blood strains	RQHR, Regina, SK, Canada
	C. albicans **knockout mutants**	
**Knockout mutant**	**Function/ Genotype description**	
*hwp1*Δ/HWP1⁺	*his3::hisG/his3::hisGleu2::tetRGAL4AD-URA3/LEU2-HWP1*	[3]
1467 (*als1*Δ/Δ)	*iro1-ura3Δ : : λimm^{434}/iro1-ura3Δ : : λimm^{434} als1saΔ/als1laΔ-URA3*[c]	[4]
1843 (*als3*Δ/Δ)	iro1-ura3Δ : : λimm^{434}/iro1-ura3Δ : :λ imm^{434} *als3laΔ/als3saΔ*-URA3[c]	[4]
HLC67 (*efg1*Δ/Δ)	*CAI4 efg1::hisG/efg1::hisG MTL*a/α[d]	[5]

[a]The full genotype at the auxotrophic markers for RSY is as follows: *his1::hisG/his1::hisGleu2::hisG/leu2::hisGarg4::hisG/arg4::hisGura3::imm434::URA3/ura3::imm434 iro1::IRO1/iro1::imm434*, as described by Sherwood and Bennett, 2008.
[b]RQHR - Regina Qu'Appelle Health Region
[c]Alleles of ALS genes are marked with LA to designate the large allele or SA for the small allele in strain SC5314.
[d]*MTL* - Mating type like

1. Sherwood, R.K.; Bennett, R.J. Microtubule Motor Protein Kar3 Is Required for Normal Mitotic Division and Morphogenesis in Candida Albicans. *Eukaryot. Cell* **2008**, *7*, 1460–1474, doi:10.1128/EC.00138-08.
2. Gillum, A.M.; Tsay, E.Y.H.; Kirsch, D.R. Isolation of the Candida Albicans Gene for Orotidine-5′-Phosphate Decarboxylase by Complementation of S. Cerevisiae Ura3 and E. Coli PyrF Mutations. *MGG Mol. Gen. Genet.* **1984**, *198*, 179–182, doi:10.1007/BF00328721.
3. Roemer, T.; Jiang, B.; Davison, J.; Ketela, T.; Veillette, K.; Breton, A.; Tandia, F.; Linteau, A.; Sillaots, S.; Marta, C.; et al. Large-Scale Essential Gene Identification in Candida Albicans and Applications to Antifungal Drug Discovery. *Mol. Microbiol.* **2003**, *50*, 167–181, doi:10.1046/j.1365-2958.2003.03697.x.
4. Zhao, X.; Oh, S.H.; Cheng, G.; Green, C.B.; Nuessen, J.A.; Yeater, K.; Leng, R.P.; Brown, A.J.P.; Hoyer, L.L. ALS3 and ALS8 Represent a Single Locus That Encodes a Candida Albicans Adhesin; Functional Comparisons between Als3p and Als1p. *Microbiology* **2004**, *150*, 2415–2428, doi:10.1099/mic.0.26943-0.
5. Lo, H.J.; Köhler, J.R.; Didomenico, B.; Loebenberg, D.; Cacciapuoti, A.; Fink, G.R. Nonfilamentous C. Albicans Mutants Are Avirulent. *Cell* **1997**, *90*, 939–949, doi:10.1016/S0092-8674(00)80358-X.

Table S2: Minimum inhibitory concentrations of EOs and Amp B against *C. albicans* background and knock-out strains.

	MIC (μg/mL)		
***C. albicans* Strains**	**CNL**	**CLV**	**Amp B**
SC5314	750 – 900	750 – 900	0.62
als1Δ/Δ (1467)	750 – 900	450 – 750	0.62
als3Δ/Δ (1843)	450 – 750	450 – 750	0.62
HLC67 (efg1Δ/Δ)	225 – 375	375 – 450	0.62
hwp1Δ/HWP1^{+}	375 – 450	375 – 450	0.31
